# Epigenetic Modifications May Regulate the Activation of the Hypopharyngeal Gland of Honeybees (*Apis Mellifera*) During Winter

**DOI:** 10.3389/fgene.2020.00046

**Published:** 2020-02-12

**Authors:** Kang Wang, Zhen-guo Liu, Zhe-guang Lin, Ling Yin, Fu-chao Gao, Guo-hong Chen, Ting Ji

**Affiliations:** ^1^ College of Animal Science and Technology, Yangzhou University, Yangzhou, China; ^2^ College of Horticulture and Plant Protection, Yangzhou University, Yangzhou, China; ^3^ Jiangsu Agri-animal Husbandry Vocational College, Taizhou, China; ^4^ Mudanjiang Branch of Heilongjiang Academy of Agricultural Sciences, Harbin, China

**Keywords:** honeybee, hypopharyngeal glands, activation, overwintering, DNA methylation

## Abstract

DNA methylation is an epigenetic modification primarily responsible for individual phenotypic variation. This modification has been reported to play an important role in caste, brain plasticity, and body development in honeybees (*Apis mellifera*). Here, we report the DNA methylation profile of honeybee hypopharyngeal glands, from atrophy in winter to arousal in the following spring, through the use of whole-genome bisulfite sequencing. Consistent with previous studies in other *Apis* species, we found low methylation levels of the hypopharyngeal gland genome that were mostly of the CG type. Notably, we observed a strong preference for CpG methylation, which was localized in promoters and exon regions. This result further indicated that, in honeybees, DNA methylation may regulate gene expression by mediating alternative splicing, in addition to silencing gene in the promoter regions. After assessment by correlation analysis, we identified seven candidate proteins encoded by differentially methylated genes, including aristaless-related homeobox, forkhead box protein O, headcase, alpha-amylase, neural-cadherin, epidermal growth factor receptor, and aquaporin, which are reported to be involved in cell growth, proliferation, and differentiation. Hypomethylation followed by upregulated expression of these candidates suggested that DNA methylation may play significant roles in the activation of hypopharyngeal glands in overwintering honeybees. Overall, this study elucidates epigenetic modification differences in honeybee hypopharyngeal glands by comparing an inactive winter state to an aroused state in the following spring, which could provide further insight into the evolution of insect sociality and regulatory plasticity.

## Introduction

Managing honeybee colonies throughout the winter has become a core agricultural issue in many countries and regions. The severity of winter conditions significantly affects successful overwintering and subsequent population growth. As insects of complex sociality, honeybees are capable of regulating their cluster temperature, normally maintaining a temperature ranging from 33 to 36°C in the presence of brood, even in the winter. However, worker bees spend considerable physical energy synthesizing and secreting royal jelly to support the larvae and the queen in cold temperatures. This task is laborious, since the workers are simultaneously responsible for temperature maintenance. However, a fraction of eggs may fail to become larvae due to low temperatures, although higher hatching rates are typically observed in larger colonies. Furthermore, the health of the surviving bees remains uncertain, since brood temperature plays a critical role in development at the larval and pupal stages (Groh et al., 2004; Jones et al., 2004; Jones et al., 2005). The cold season also reduces nectar secretion from nectariferous plants and limits the foraging behavior of the workers. The higher rate of energy consumption combined with lower production during winter results in maladapted reproductive behavior of queens during the winter.

**Figure 1:**
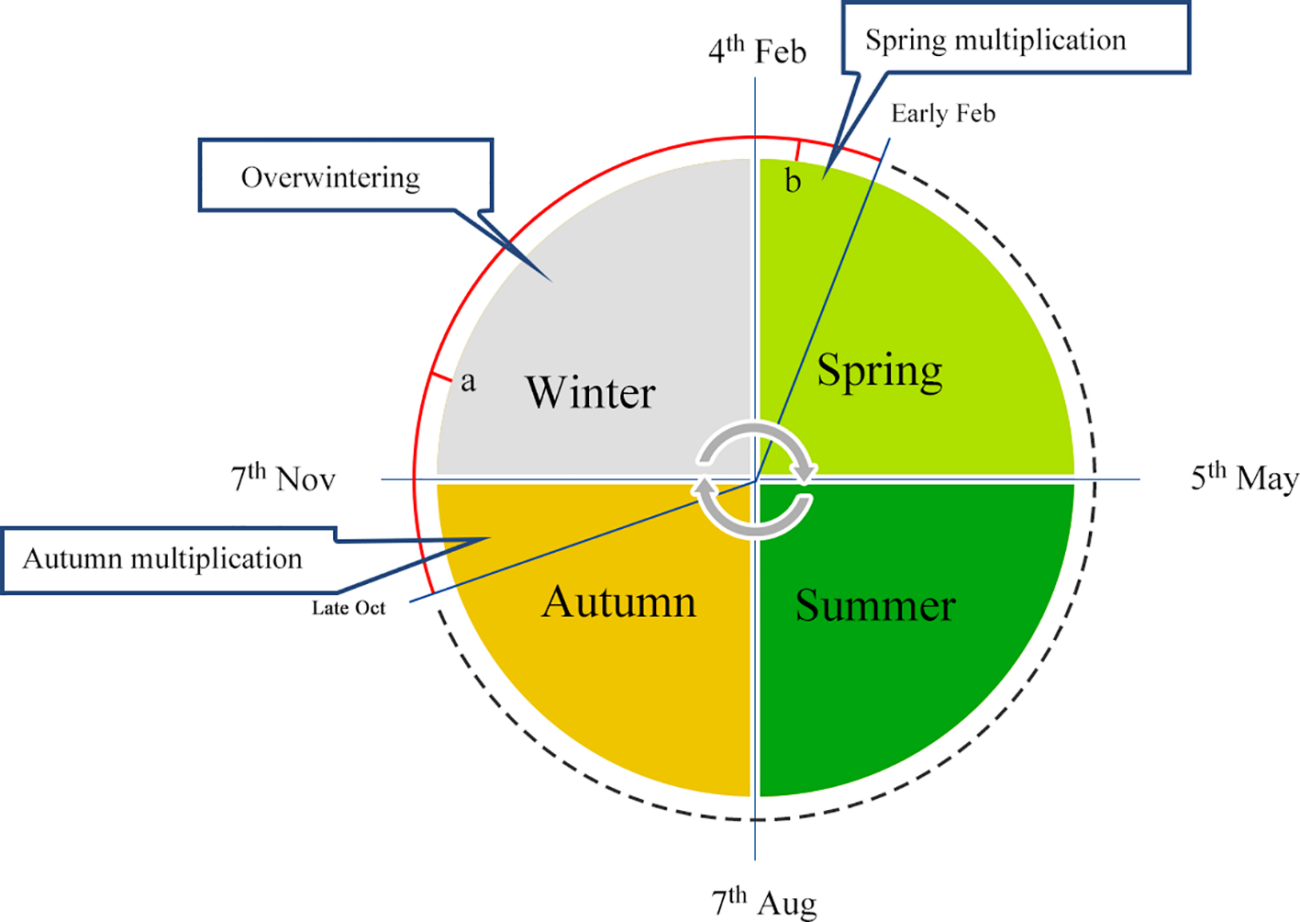
Annual schedule of overwintering management. Honeybee overwintering typically occurs from October to the following March, and samples collected at points a and b represent winter and spring bees, respectively.

In China, a series of effective manual interference solutions have been adopted by expert beekeepers for overwintering management, which mainly involve caging the queen to prevent reproduction. In the mid-lower reaches of the Yangtze River, the temperature during winter is moderate, and a temperature of −10°C is typically recorded for only two weeks, allowing queens to continue laying eggs during this season. Generally, colonies are fed with supplemental pollen and syrup at the end of October before the queen is stimulated to lay eggs to obtain a sufficient quantity of overwintering bees. Queens are then restricted to an *in-situ* cage, located in the warm hub of the cluster, in order to prevent an inclination to rear the brood. As shown in **Figure 1**, the queen is caged in place, and worker bees are kept motionless in the hive of the overwintering cluster for a long period (around points a to b, **Figure 1**) at low temperatures. Subsequently, the queen is released, and egg-laying behavior is promoted. The queen and larvae are then fed by energetic worker bees, causing colonies to become a swarm. Worker honeybees that survive the winter are not burdened with foraging or larvae feeding from late November or early December through to the early days of the following February, since the queen is restricted in movement. Therefore, workers maintain desirable conditions for the colony during the process of overwintering and lay the foundation for reproduction in spring the following year, when colonies rapidly multiply.

The hypopharyngeal glands are essential excretory glands that secrete royal jelly proteins, which play an important role in the nutrition and caste system of honeybees especially (Kamakura, 2011). In previous studies, we investigated the hypopharyngeal glands of worker honeybees involved in the commercial overwintering management described above, through morphologic and large-scale proteomics approaches. We did not find well-developed organelles in inactive hypopharyngeal glands during winter. Instead, the acinar cells of worker honeybees during spring contained more mature organelles, and protein biosynthesis was strengthened, including that in ribosomes, endoplasmic reticula, and extracellular ducts, which synthesize and transport major royal jelly proteins in the cells of the hypopharyngeal glands (Wang et al., 2018). Plasticity is a typical characteristic of social insects, and the change in status of hypopharyngeal glands from a suppressed to activated cycle, during which foragers are forced to revert to hive-tasks, is an example of a highly flexible physiological reversion (Knecht and Kaatz, 1990; Deseyn and Billen, 2005). Multiple lines of evidence suggest that DNA methylation, a process of gene silencing that is heritable across cell divisions, may serve as the underlying mechanism for the evolution of plasticity. In particular, several studies on honeybees have highlighted the potential importance of methylation for understanding plasticity that controls reproduction in workers (Kucharski et al., 2008; Duncan et al., 2016). Therefore, we hypothesized that changes in stimulus signals and the environmental intervention (larvae or nutrition) induced by commercial overwintering management may possibly contribute to arousal of hypopharyngeal glands in the spring *via* epigenetic processes. Here, we report DNA methylation patterns involved in the activity of hypopharyngeal glands in overwintering honeybees, focusing on mRNA expression for seven overlapping differentially methylated genes (DMGs). Our results suggest that hypopharyngeal glands may be activated through certain candidate pathways, including the epidermal growth factor receptor-mediated signaling pathway. Overall, this study elucidates epigenetic modification differences in honeybee hypopharyngeal glands, comparing an inactive winter state to an aroused state in the following spring, and will provide further insight into the evolution of insect sociality and regulatory plasticity.

## Materials and Methods

### Honeybee Preparation

To obtain similar genetic backgrounds, honeybees (*A. mellifera*) were generated from a single drone-inseminated queen. Six healthy colonies were raised at the Honeybee Research Institute, Yangzhou University, Yangzhou, China. The colonies were maintained according to standard commercial overwintering beekeeping practices in China (Wang et al., 2018). To ensure that sampled worker bees were of the same age, the thoraxes of thousands of 1-day old individuals (mid-month in November) were painted with pigment and then reintroduced into their original hives.

### Honeybee Collection

Bees were collected at two key time points in the winter–spring schedule, as described below. Worker bees in the winter bee (WB) group were kept flightless and restricted to hives throughout the long period of low temperature. Here, we defined winter bees as those sampled at the coldest period, based on historical experience, generally during early January in China; therefore, winter bees were collected on January 8, 2015. To produce overwintering bees in the feeding phase, queens were set free on February 8, 2015 (day 1) and stimulated with diluted syrup to lay numerous eggs. We observed that a handful of larvae hatched on February 12 (day 5). Subsequently, large numbers of larvae began to hatch, and spring worker bees entered a period of larval feeding on days 6 and 7. Therefore, by the time of collection (day 8, February 15), bee physiology had already shifted to “nurse bees”. Only worker bees exhibiting obvious feeding behavior were sampled and considered as spring bee (SB) subjects.

Two conditions must be met for colonies at two key time-points in the winter–spring schedule, respectively. First, queens had to be confined *in situ* until artificial release. A push-type cage, equipped with a mesh sieve to exclude queens, is usually chosen to limit movement range. However, mutual crowding of honeybees in colonies enables easy accidental unlocking of excluder buckles, which could fail to prevent egg laying. The presence of larvae is considered a stimulus for hypopharyngeal gland activation (Huang and Otis, 1989). Accidents like this are considered mismanagement in commercial overwintering practice and did not occur during this study. Second, observation of worker bee feeding behavior is required to ensure successful activation of hypopharyngeal glands. A cold spell in a relatively warm spring may prevent eggs from becoming larvae, which could prevent worker bees from driving feeding behaviors and lead to incomplete hypopharyngeal gland activation. In the present study, we strictly focused on hypopharyngeal gland change from inactivity in winter to arousal in the following spring. Marked workers were sampled randomly following the strategy described. The worker bees were anesthetized with carbon monoxide, and their heads were fixed on a smooth wax dish. Chitin in the head was dissected using a knife blade to cut along the edge from one canthus to another, and the exposed hypopharyngeal glands were clipped out with forceps under a microscope. Three biological replicates were performed for each of the WB and SB groups.

### Methylation Library Preparation and Sequencing

Three biological replicates of the pooled sample (30 hypopharyngeal glands from each hive) were prepared for further analyses. A Universal Genomic DNA Extraction Kit (TaKaRa, Tokyo, Japan) was used to extract DNA from hypopharyngeal gland samples. For library construction, DNA was fragmented by sonication using a Bioruptor (Diagenode, Liège, Belgium) to a mean size of approximately 250 bp, followed by blunt-ending, dA addition to the 3′-end, and adaptor ligation (with the addition of methylated adaptors to protect DNA from bisulfite conversion), according to the manufacturer's instructions (DV811A). Ligated DNA was subjected to bisulfite conversion using the EZ DNA Methylation-Gold kit (Zymo Research, Irvine, CA, USA). Fragments with different insert sizes (300−500 bp) were excised from the same lane of a 2% TAE agarose gel. Products were purified using a QIAquick Gel Extraction kit (Qiagen, Hilden, Germany) and amplified by PCR. The 50 μl reaction volume contained 25 μl FailSafe™ PCR Premix E, 1 μl TruSeq DNA Methyl Forward (Illumina Inc., San Diego, CA, USA), 1 μl Index PCR Primer, 0.5 μl FailSafe™ PCR Enzyme, and 22.5 μl di-tagged DNA. Amplification was conducted as follows: 95°C for 1 min; 10 cycles at 95°C for 30 s, 55°C for 30 s, and 68°C for 3 min; and 68°C for 7 min. Finally, sequencing was performed using the HighSeq2000 platform (OEBiotech, Shanghai, China).

### Bioinformatics Analysis

Data filtering included removal of adaptor sequences, contamination, and low-quality reads from the raw reads. After filtering, clean data were mapped to the reference genome (ftp://ftp.ncbi.nlm.nih.gov/genomes/all/GCF/000/002/195/GCF_000002195.4_Amel_4.5/GCF_000002195.4_Amel_4.5_genomic.fna.gz) using the whole-genome bisulfite sequencing MAPping program (BSMAP) (Xi and Li, 2009). Duplicate reads were then removed and the mapping rate and bisulfite conversion rate were calculated for each sample. Methylation levels were determined by dividing the number of reads covering each cytosine (mC) by the total reads covering each cytosine, which were also equal to the mC/C ratio at each reference cytosine. All mC sites were divided into specific gene features. The C site methylation was determined by a binomial distribution test (Sun et al., 2014), and different methylation sites were defined across two groups with a false discovery rate (FDR) lower than 0.05 (Schultz et al., 2012). Differential methylation regions (DMRs) were defined as regions that contained no less than three distinct methylated sites, where the difference in the degree of methylation between sites was higher than 0.1 and the *p-*value obtained by Fisher's exact test was lower than 0.05.

### Correlations Between Methylation and mRNA Expression

To further screen functional genes involved in honeybee hypopharyngeal gland activation regulated by epigenetics, we analyzed the expression of select functional genes by qRT-PCR. Methylation has been reported to regulate gene expression by preventing transcription. Hence, we focused on the negative correlation between DMGs and differentially expressed genes. Thus, candidate genes for further analyses were selected based on a pattern of hypomethylation and overexpression or hypermethylation and downregulation. The qRT-PCR data were normalized to *β*-actin expression using the 2^-△△Ct^ method, and an independent-sample t-test was adopted to evaluate significant differences (with *P* < 0.05 as the threshold) between the SB and WB groups.

## Results

### DNA Methylation Mapping and Patterns

**Figure 2:**
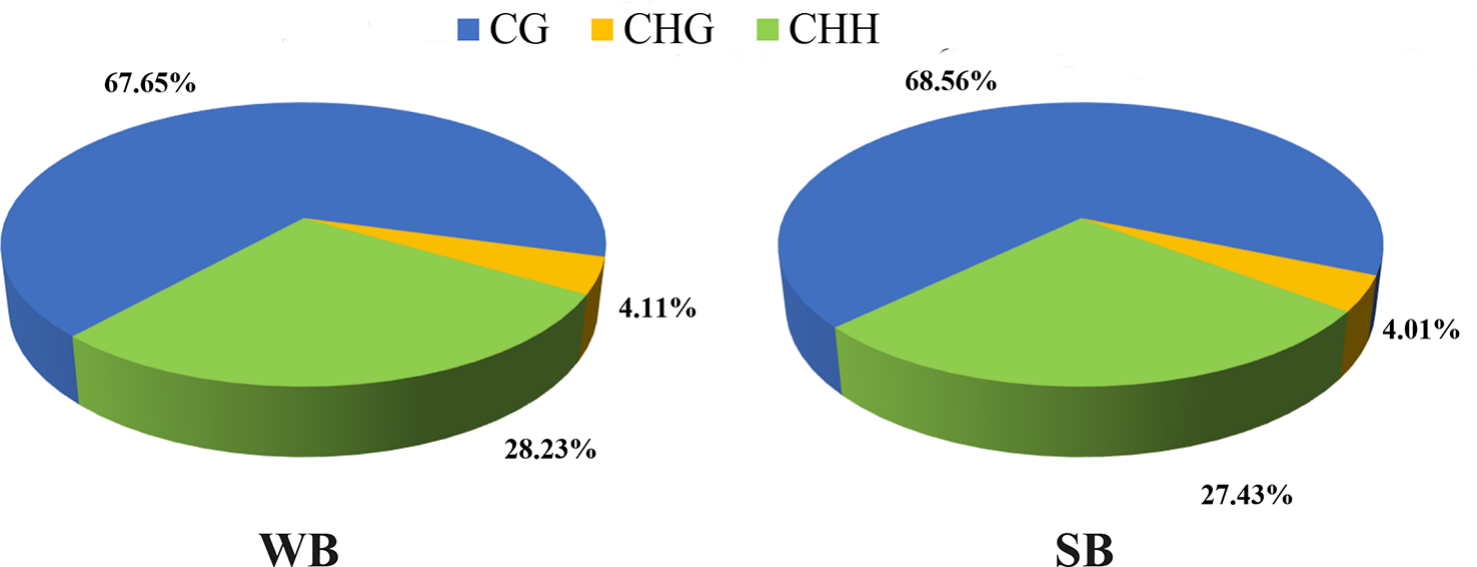
The average ratio of DNA methylation types in the genomes of WB and SB. WB, Winter Bee. SB, Spring Bee. H = A, C, or T. The blue, green, and yellow colors represent mCG, mCHG, and mCHH, respectively.

After filtering, an average of 5.93 G clean bases and 64 million clean reads, distributed ubiquitously among chromosomes, were identified for each group. We subjected 79–82% of the mapped reads to further analyses. In each WB and SB group, 0.56% of all genomic C sites were methylated, on average (**Table 1**). Similar to that observed in other species, methylation in honeybees occurs in three sequence contexts: CG, CHG (where H is A, C or T), and CHH. We found the overall levels of genome-wide methylated cytosine to be 67.65% CG, 4.11% CHG, and 28.23% CHH methylated C sites in the WB group, and 68.56% CG, 4.01% CHG, and 27.43% CHH methylated C sites in the SB group (**Figure 2**). To gain a better understanding of the methylation information in the two groups, a sequence preference analysis was conducted, including the global distribution pattern of methylation levels for various types of methylations (**Supplementary Figure S1**) and the relationship between sequence context and methylation preference (**Supplementary Figure S2**). We found that the methylation levels of all sample presented a broad similarity and that most hypermethylation were of the CG type. Furthermore, we analyzed the percentage of methylation for all possible 9-mer sequences where the mC was in the fourth position. The sequence preference analysis showed the mC sites were in a hypermethylation state, the CAG was the most common sequence motif in the CHG mC sites, and CAT and CAA were discovered in the CHH mC sites.

**Table 1:** Whole genome DNA bisulfite sequencing data.

Group	Sample	Clean Data (Gb)	Clean Reads (Millions)	Mapping Rate (%)	Bisulfite Conversion Rate (%)	Total_mC (%)
WB	WB1	5.8	64	79.47	99.56	0.66
	WB2	6	67	80.99	99.59	0.53
	WB3	5.89	65	81.78	99.65	0.49
SB	SB1	5.98	66	79.47	99.53	0.63
	SB2	5.94	66	79.47	99.57	0.56
	SB3	5.34	59	82.39	99.63	0.5

Clean Date (Gb), number of clean bases throughout the sequence. Clean Reads (Millions), number of clean reads. Mapping rate (%), number of clean reads corresponding to the reference genome relative to total clean reads. Bisulfite Conversion Rate (%), number of clean reads matched to the reference genome relative to amount of methylation of clean reads matched to the reference genome. Total mC (%), number of clean reads matched to the reference genome relative to the amount of methylated cytosine within the clean reads matched to the reference genome.

### Methylation Levels of DNA in Regions of Various Functions

**Figure 3:**
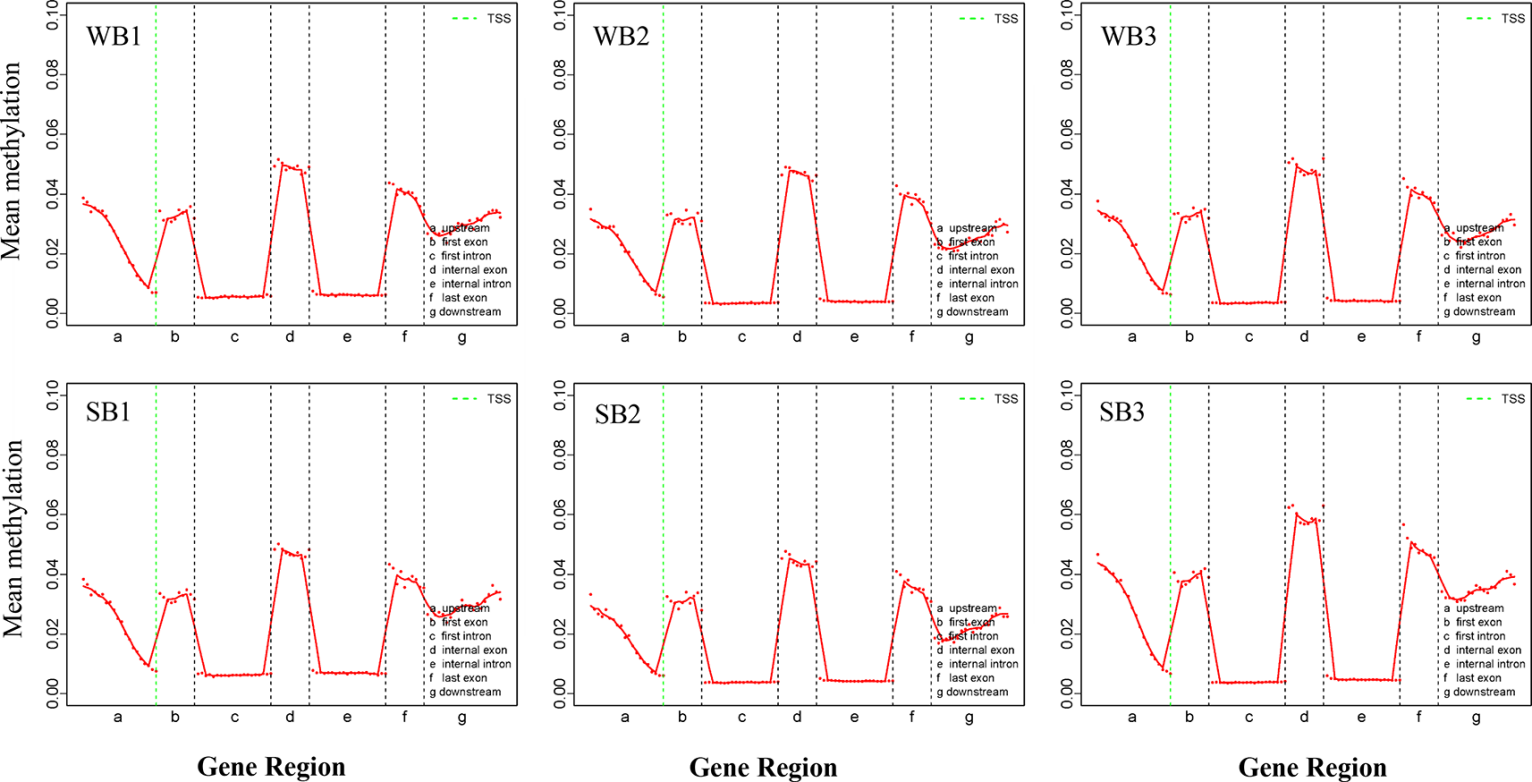
The trend of mCG type distribution across different functional regions. WB, Winter Bee; SB, Spring Bee. The abscissa represents the different regions of gene functional elements. The dotted green line denotes the transcriptional start site. The ordinate represents the methylation levels of mCG.

All mCs were classified on the basis of the following genetic characteristics: 3′ UTR, intron, exon, and 5′ UTR; in addition, the promoter and methylation degree were evaluated in the above-mentioned areas. The levels and patterns of DNA methylation in various chromosomal features (gene, exon, and intron promoters) were broadly similar across the WB and SB groups (**Figure 3**, **Supplementary Figures S3** and **S4**). Methylation levels of the CG type showed significant changes in different functional regions. Specifically, in the upstream region (< 2 kb before the transcription start site), DNA methylation levels decreased continuously, reaching the lowest point at sites near the transcription start site (TSS). After TSS, methylation levels exhibited abrupt waves between regions of inner exons and introns, and the latter always maintained hypomethylation (**Figure 3**). The methylation levels of CHG and CHH type were similar and below 0.01% and stable in each functional element (**Supplementary Figures S3** and **S4**).

### DMR Identification and Enrichment Analysis of Methylated Gene Functions

In our study, 428 DMRs were detected between the WB and SB groups. In total, 372 DMR-associated genes were identified (**Supplementary Table S1**). These DMGs were annotated into gene functional elements, including those upstream (< 2 kb before the TSS), in the gene body, and downstream (< 2 kb after transcription termination site). We did not analyze the methylation of distal intergenic regions since methylation in these regions is usually considered inconsequential to gene expression (Jaenisch and Bird, 2003; Jones, 2012). Relative to the upstream and downstream regions, gene bodies (68.07%) exhibited the highest methylation levels in hypopharyngeal gland tissues during overwintering (**Supplementary Table S1**).

We observed a negative correlation between methylation and gene expression, and seven eligible genes emerged after filtration, providing a better understanding of the biochemical changes occurring in the hypopharyngeal glands during overwintering (**Supplementary Table S2**). Interestingly, all candidate genes exhibited upregulated expression and a hypomethylated pattern in spring, including aristaless-related homeobox gene (ARX, GB50532), forkhead box protein O (FOXO, GB48301), alpha-amylase (*α*-amylase, GB49854), headcase (GB47931), neural-cadherin (N-cadherin, Gene ID: 102655737), epidermal growth factor receptor (EGFR, GB54477), aquaporin (GB41225), and an unrecognized gene with unknown functions (uncharacterized LOC551079, GB47623) (**Supplementary Table S2**). Among these seven genes, only the aquaporin gene was demethylated in the gene promoter region, while most candidate DMG sites were located in the gene body.

## Discussion

The long lifespan of worker bees is a fundamental requirement for successful overwintering, and can be further extended through reduction of unnecessary metabolic activity by manual interference.

Overwintering management with caged queens prevents reproductive behavior and feeding behavior in worker honeybees, requiring the workers to suspend other activities temporarily and focus solely on maintaining hive temperature through generation of body heat. This phenomenon could be described as diapause in a superorganism, where a low rate of basal metabolism is maintained to ensure honeybee survival. This efficient overwintering management has been implemented for many years in north and central China, regions where colony populations grow at high rates. The regulatory mechanism by which the hypopharyngeal gland controls arousal during the spring after an inactive period is an important area of study. We have previously demonstrated that larvae and nutrients stimulate activation of hypopharyngeal glands, which is accompanied by remodeling of organelles and secretion of intracellular proteins for overwintering management (Wang et al., 2018). Here, we report the DNA methylation profile of honeybee hypopharyngeal glands, from atrophy in winter to arousal in the following spring, using whole-genome bisulfite sequencing. We uncovered several DMR-related genes involved in a functional signaling pathway, suggesting that methylation may play an important role in the activation and construction of hypopharyngeal glands.

Genomic methylations play an essential role in regulating metabolism and growth and are particularly important for establishing caste distinctions. However, the methylation levels of DNA among honeybees are significantly lower than those in other mammals. Thus, consistent with previous studies (Foret et al., 2012), we found that methylation levels for the hypopharyngeal gland genome were maintained at a low state, and the majority were of type CG (68%). Intriguingly, compared to the low level and uniform distribution of each functional element comprising the CHG and CHH types, the CG type showed a distinctive and regular wave in every functional element, and methylation occurred solely in promoters associated with gene expression and exon regions related to alternative splicing (**Figure 3**). Methylated genes had expression differences due to transcriptional inhibition in the promoter region. In social insects, however, methylation of the gene body may regulate gene expression by mediating alternative splicing (Lyko et al., 2010; Bonasio et al., 2012; Foret et al., 2012). Here, the strong localization of methylation to the vicinity of splicing sites in exons that we observed may be related to alternative splicing, as previously reported (Foret et al., 2012; He et al., 2017). Gene expression is also regulated by alternative splicing in addition to methylations located in the promoter region. Our work provides further support for the supposition that methylation in the gene body region exerts a more complex role than merely suppressing expression. This is a valuable and meaningful observation that merits further investigation.

In the present study, gene expression patterns may have been altered upon DNA methylation during the activation of hypopharyngeal glands over the winter-spring period. Hence, the selection of seven potential functional genes enabled us to better understand the biochemical activation of hypopharyngeal glands in overwintering worker bees. For example, the aristaless-related homeobox gene, forkhead box protein O, alpha-amylase, headcase, and neural-cadherin were found to be significantly hypomethylated and upregulated after activation. ARX belongs to the family of aristaless-related genes, which may play a critical role in regulating central and peripheral nervous system development in vertebrate embryogenesis (Simeone et al., 1994; Friocourt et al., 2006). FOX proteins are widely known as transcription factors that play important roles in regulating the expression of genes involved in cell growth, proliferation, differentiation, and longevity (Galili et al., 1993; Tuteja and Kaestner, 2007a; Tuteja and Kaestner, 2007b). The FOXO subfamily of Forkhead transcription factors has been identified as a direct target of phosphoinositide 3-kinase-mediated signal transduction, which is associated with molecular mechanisms controlling the division and differentiation of cells (Birkenkamp and Coffer, 2003). Intriguingly, FOXO acts as a contributor to juvenile hormone (JH) synthesis, which modulates multiple signaling pathways, and leads to diverse caste polyphenism in social insects, which may manifest as differences in size, morphology, and physiology (Kamakura, 2011; Alvarado et al., 2015; Corona et al., 2016). Alpha-amylase, which is considered a key enzyme for carbohydrate metabolism, is known to be associated with the development of hypopharyngeal glands and is possibly induced by a nutritional rather than a larval stimulus. Upregulation of headcase genes has been reported to precede imaginal cell re-entry into the mitotic cell cycle and persist until final cell divisions (Weaver and White, 1995). Loss of headcase expression did not affect imaginal cell growth, but it interfered with the ability of the imaginal primordia to undergo proper differentiation during metamorphosis in *Drosophila melanogaster* (Resende et al., 2013); therefore, the headcase gene is considered to be an imaginal-specific gene required for adult morphogenesis. Previous studies demonstrated that N-cadherin signaling affects fundamental cellular processes such as proliferation, differentiation, and, in particular, cell morphology and migration, which in turn affects tissue morphogenesis and structure (Kashima et al., 1999; Wheelock and Johnson, 2003; Niimi et al., 2013).

Moreover, we have reported two additional target genes of DNA methylation that may be important for activation of hypopharyngeal glands: the epidermal growth factor receptor (EGFR) and the aquaporin genes, which were found to be hypomethylated and upregulated. EGFR has been widely reported to encode a transmembrane protein that is highly expressed in epidermal and stromal cells. It plays an important role in cell division by regenerating the epidermis from stromal cells to initiate morphogenesis upon activation by its specific ligands, such as transforming growth factor *α* and epidermal growth factor (Yarden and Schlessinger, 1987; Oda et al., 2005). Once bound, phosphorylation is stimulated and downstream pathways, such as the mitogen-activated protein kinase, serine-threonine protein kinase, and c-Jun N-terminal kinase pathways, are then activated, which regulate DNA synthesis, growth, cell proliferation, differentiation, and many other vital cellular processes (Jorissen et al., 2003). In insects, such as honeybees and fruit flies, EGFR systems have been shown to play roles in caste determination (Kamakura, 2011), as well as in controlling the size of worker ants (Alvarado et al., 2015). The FOXO gene, as described above, may also be positively regulated by EGFR (Corona et al., 2016). Hence, by summarizing the regulatory networks of the functional genes involved in caste polyphenism in social honeybees, we speculated that stimuli such as larger spaces and high nutritional status increase the levels of insulin-like proteins and activate insulin receptors and downstream kinases, which then interact with the EGFR pathway to regulate activity of the FOXO transcriptional factor. Further, non-phosphorylated FOXO promotes synthesis of JH, which exerts multiple effects on various signaling pathways, leading to different caste traits and physiology polyphenism. In previous reports, the JH titer increased in February (165-day old) as compared to that in December and January (100-day and 130-day old) (Fluri et al., 1977), which is consistent with our prediction. Although the study did not specifically mention whether caged-queen overwintering management was utilized, bee physiology in winter must have been maintained at hypometabolism compared to that in the active spring stage. Given the current evidence, we suggest that DNA methylation regulates cell signaling genes, including epidermal growth factor receptor and forkhead box protein O, to facilitate the re-development of hypopharyngeal glands, which would otherwise atrophy in winter.

Aquaporins are members of the major intrinsic protein family, which is expressed by almost all living organisms. Some aquaporins transport small solutes, such as glycerol (Zeidel et al., 1992; Kataoka et al., 2009; Tomkowiak and Pienkowska, 2010). In honeybee crops, water from nectar is absorbed *via* aquaporins to increase the sugar concentration (Serrão et al., 2014). However, multiple lines of evidence indicate that these proteins are involved in numerous cellular processes. For instance, Aquaporin 1 is highly expressed in the proliferating microvessels of many tumors (López-Campos et al., 2011; Galán-Cobo et al., 2016), and aquaporin 1 has been confirmed to have a fundamental role in angiogenesis and cell migration by comparing phenotypes between aquaporin-null and wild-type mice (Saadoun et al., 2005). These results are most likely due to the role of aquaporins play in facilitating changes to the cell volume and activation of necessary channels or transporters. It is well established that royal jelly secreted from the hypopharyngeal glands is conveyed through the extracellular ducts, which are considered necessary functional organs, to the large excretory duct. According to previous transmission electron microscopy studies on the structure of hypopharyngeal glands from overwintering honeybees, extracellular ducts were found to be degraded in the inactive winter stage, and then rebuilt in the following spring to convey royal jelly to support increased numbers of larvae. As previously speculated, additional unexpected roles for aquaporins will undoubtedly be discovered in the future (Galán-Cobo et al., 2016). Given that aquaporins have versatile channel functions, it seems reasonable to presume that they may be involved in rebuilding extracellular ducts in the hypopharyngeal gland cells of spring honeybees, which could be a new royal jelly channel beyond water permeability.

In this work, we identified several potential functional genes related to arousal of hypopharyngeal glands from inactivity in winter to activity in the following spring *via* epigenetic processes, which could provide further insight into the evolution of insect sociality and regulatory plasticity. However, despite accumulating knowledge on lots of genes related to honey bee diverse phenotype and social behaviors, it remains unclear whether these genes actually work, in part because of the scarcity of genetic manipulation methods and availability of particular cell lines for application to honey bees. Therefore, further exploration will be necessary to verify the hypothesis in our study in the not too distant future.

## Data Availability Statement

The datasets generated for this study can be found in the SRA (Sequence Read Archive) database (SRA accession number: SRP212812).

## Author Contributions

The manuscript was written through contributions of all authors. All authors have given approval to the final version of the manuscript. Conceived and designed the experiments: KW, Z-gLiu, TJ, and G-hC. Performed the experiments: KW and Z-gLin. Analyzed the data: KW, G-hC, and LY. Raised bees and collected samples: Z-gLiu, LY, and F-cG. Wrote the paper: KW and Z-gLin.

## Funding

This work was supported by grants from the Natural Science Foundation of Heilongjiang province (No. C2017062) and Jilin province (No. 20180101022JC). Modern Agroindustry Technology Research System (CARS-45-SYZ6) and the Technology Research and Development Program of Jiangsu Province, China (BE2018353).

## Conflict of Interest

The authors declare that the research was conducted in the absence of any commercial or financial relationships that could be construed as a potential conflict of interest.

## Supplementary Material

Figure S1 Global distributing pattern of methylation degrees for various types of methylations. WB, Winter Bee. SB, Spring Bee. The red, blue and green colors represent mCG, mCHG, and mCHH respectively. This picture shows relationship between methylation levels and percentage of total mC.

Figure S2 Relationship between sequence context and methylation preference. WB, Winter Bee. SB, Spring Bee. The abscissa represents all possible 9-mer sequences where the mC was in the fourth position and the ordinate represents the percentage of bases.

Figure S3 The methylation levels of CHH type in various functional regionsThe trend of mCHH type distribution across different functional regions. WB, Winter Bee; SB, Spring Bee. The abscissa represents the different regions of gene functional elements. The dotted green line denotes the transcriptional start site. The ordinate represents the methylation levels of mCHH.

Figure S4 The methylation levels of CHG type in various functional regionsThe trend of mCHG type distribution across different functional regions. WB, Winter Bee; SB, Spring Bee. The abscissa represents the different regions of gene functional elements. The dotted green line denotes the transcriptional start site. The ordinate represents the methylation levels of mCHG.
