# Supplementary figures and images for: Epigenetic Modifications May Regulate the Activation of the Hypopharyngeal Gland of Honeybees (Apis Mellifera) During Winter
Source: Front Genet. 2020 Feb 12;11:46. doi: 10.3389/fgene.2020.00046 (PMC7029738; doi:10.3389/fgene.2020.00046)

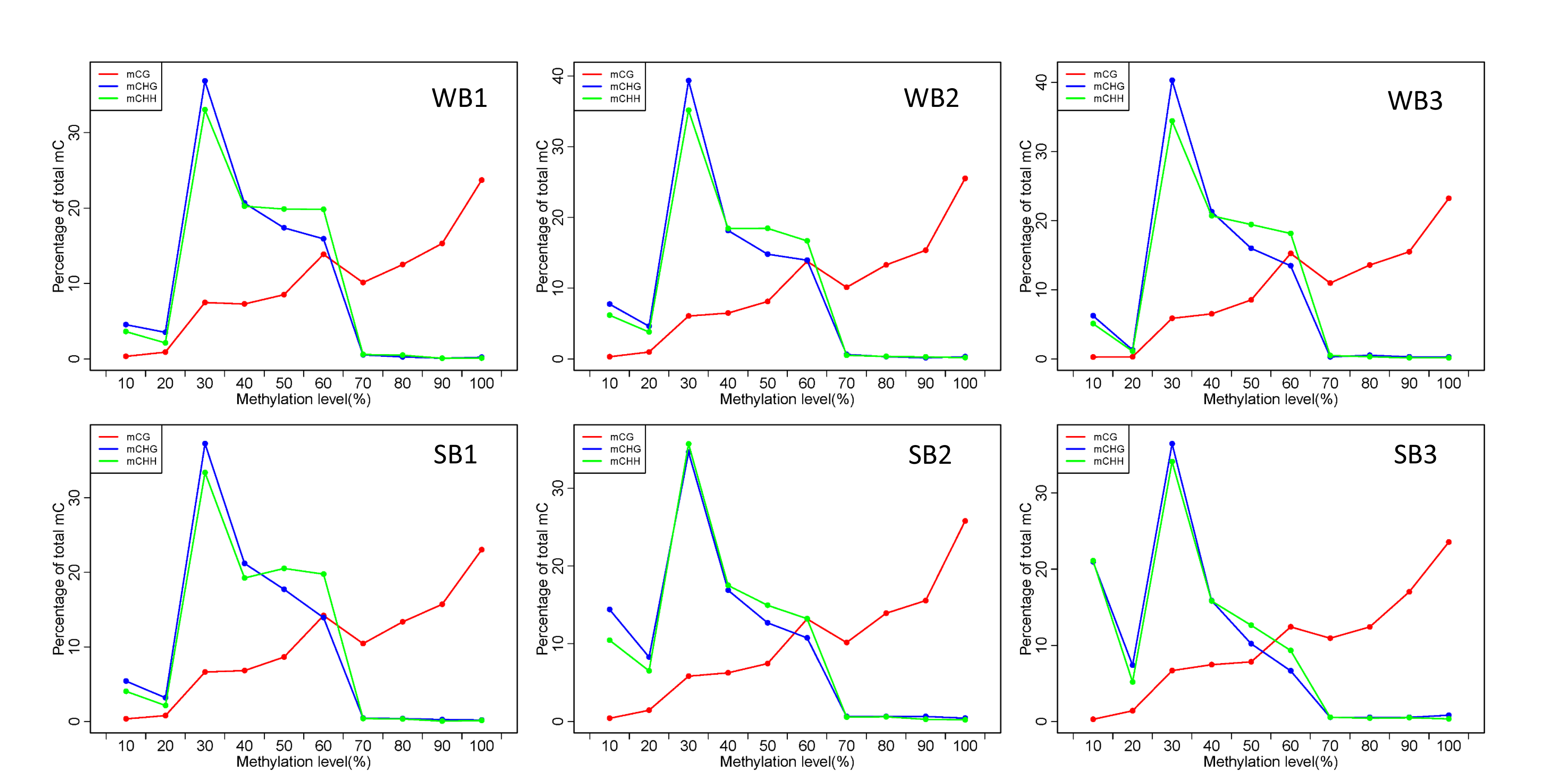

Supplement: Figure S1 — Global distributing pattern of methylation degrees for various types of methylations. WB, Winter Bee. SB, Spring Bee. The red, blue and green colors represent mCG, mCHG, and mCHH respectively. This picture shows relationship between methylation levels and percentage of total mC. [file Image_1.tif]

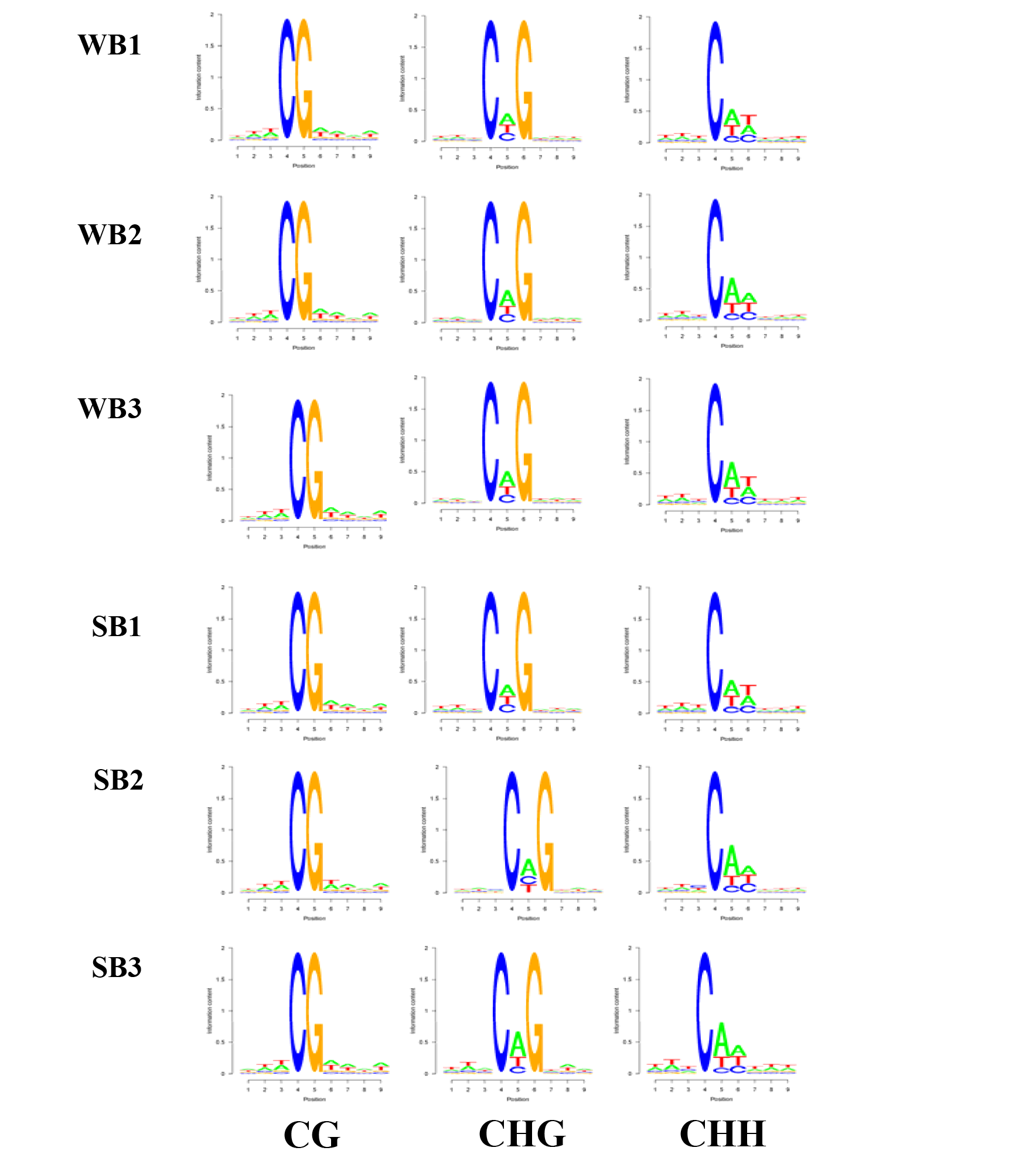

Supplement: Figure S2 — Relationship between sequence context and methylation preference. WB, Winter Bee. SB, Spring Bee. The abscissa represents all possible 9-mer sequences where the mC was in the fourth position and the ordinate represents the percentage of bases. [file Image_2.tif]

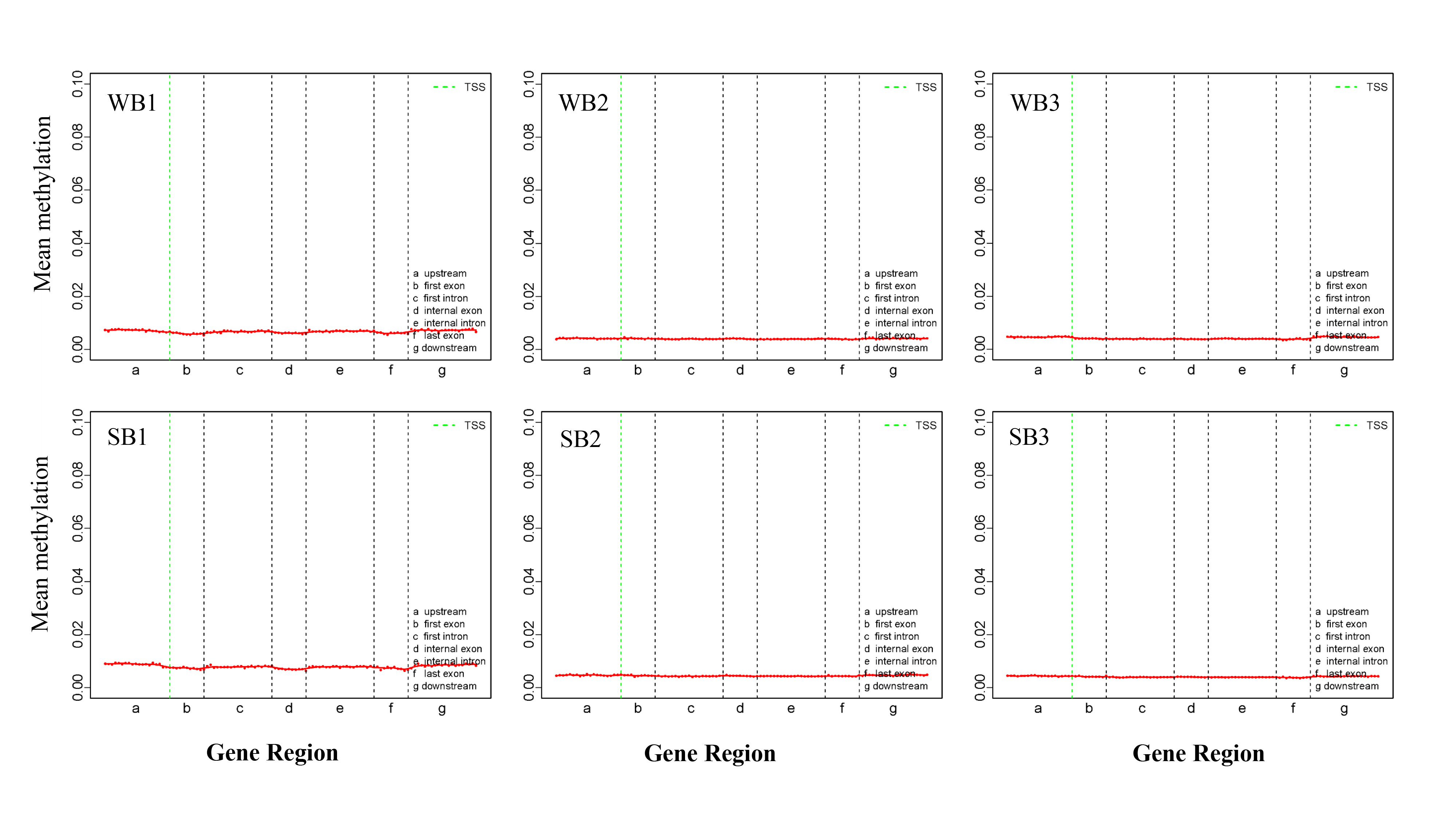

Supplement: Figure S3 — The methylation levels of CHH type in various functional regionsThe trend of mCHH type distribution across different functional regions. WB, Winter Bee; SB, Spring Bee. The abscissa represents the different regions of gene functional elements. The dotted green line denotes the transcriptional start site. The ordinate represents the methylation levels of mCHH. [file Image_3.tif]

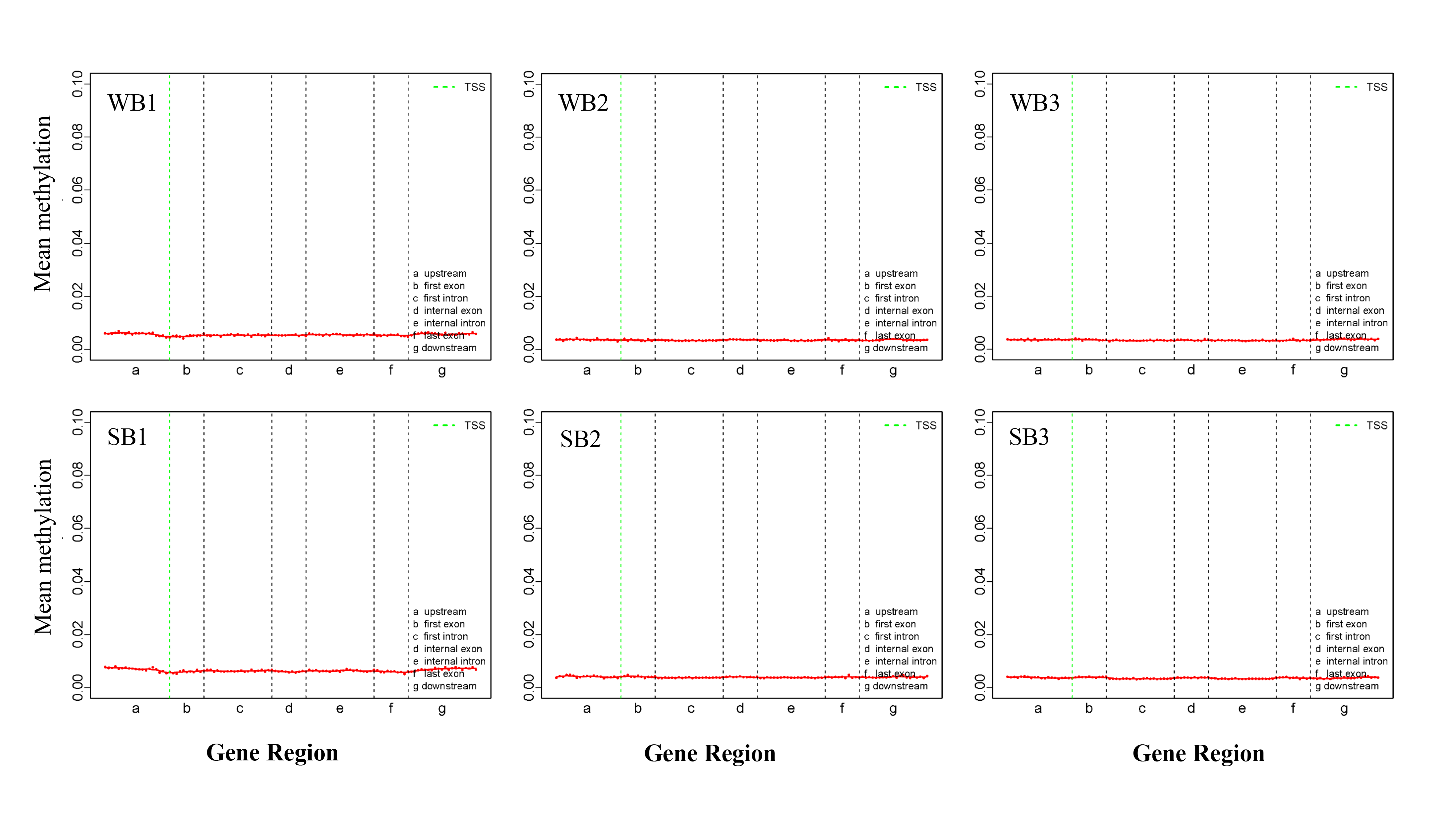

Supplement: Figure S4 — The methylation levels of CHG type in various functional regionsThe trend of mCHG type distribution across different functional regions. WB, Winter Bee; SB, Spring Bee. The abscissa represents the different regions of gene functional elements. The dotted green line denotes the transcriptional start site. The ordinate represents the methylation levels of mCHG. [file Image_4.tif]
